# Factors Affecting Secondary Traumatic Stress of Nurses Caring for COVID-19 Patients in South Korea

**DOI:** 10.3390/ijerph18136843

**Published:** 2021-06-25

**Authors:** Mee Sun Lee, Sujin Shin, Eunmin Hong

**Affiliations:** College of Nursing, Ewha Womans University, Seoul 03760, Korea; ms5723460@naver.com (M.S.L.); ssj1119@ewha.ac.kr (S.S.)

**Keywords:** COVID-19, nurses, compassion fatigue, secondary traumatic stress

## Abstract

The secondary traumatic stress (STS) of nurses caring for COVID-19 patients is expected to be high, and it can adversely affect patient care. The purpose of this study was to examine the degree of STS of nurses caring for COVID-19 patients, and we identified various factors that influence STS. This study followed a descriptive design. The data of 136 nurses who had provided direct care to COVID-19 patients from 5 September to 26 September 2020 were collected online. Hierarchical regression analysis was conducted to identify the factors influencing STS. Participants experienced moderate levels of STS. The regression model of Model 1 was statistically significant (F = 6.21, *p* < 0.001), and the significant factors influencing STS were the duration of care for patients with COVID-19 for more than 30 days (β = 0.28, *p* < 0.001) and working in an undesignated COVID-19 hospital (β = 0.21, *p* = 0.038). In Model 2, the factor influencing STS was the support of a friend in the category of social support (β = −0.21, *p* = 0.039). The nurses caring for COVID-19 patients are experiencing a persistent and moderate level of STS. This study can be used as basic data to treat and prevent STS.

## 1. Introduction

Infectious diseases caused by coronavirus variants have afflicted people all over the world, beginning with severe acute respiratory syndrome (SARS) in 2003, Middle East respiratory syndrome (MERS) in 2012, and coronavirus disease 2019 (COVID-19) in late 2019. In particular, as people continued to be infected with COVID-19, the World Health Organization (WHO) declared it a global pandemic [1]. As 24 of May 2021, there were about 165 million confirmed cases of COVID-19 around the world, and about 3.4 million people have died from COVID-19 [2]. COVID-19 has an average transmissibility like other existing infectious diseases, but a therapeutic drug has not been developed so far; thus, it continues to infect people with an increasing trend [1]. Accordingly, “social distancing” has been adopted in South Korea to prevent the spread of COVID-19 in the country. Nurses play major roles in preventing the spread of COVID-19 infection by screening and classifying patients, as well as nursing COVID-19 patients [3]. 

Nurses risk contracting the disease and take care of the rapidly increasing number of COVID-19 patients for nearly 24 h daily. Nurses experience various mental stresses in the process of caring for these patients. According to on-site records of nurses who directly cared for COVID-19 patients in Korea, nurses understood patients’ fears and showed empathy for the patients. However, they complained of a lack of medical resources, fatigue due to high intensity of work, the discomfort in wearing personal protective equipment (PPE), difficulties in nursing performance, anxiety from concerns regarding family, and fears about transmission and infection [4]. In addition, nurses who cared for COVID-19 patients in China also complained of anxiety for their families and patients, fear, and lethargy and fatigue due to high-intensity work [5]; further, they complained of loss of appetite, indigestion, insomnia, numbness, a desire to cry sometimes, suicidal thoughts, etc. [6]. According to the Korean Society for Trauma Stress [7], healthcare workers during an infectious disease outbreak can experience emotional stress, exhaustion, fear, anxiety, anger, helplessness, and decreased judgment. Secondary traumatic stress (STS), described as experiencing the patient’s trauma, may occur among nurses; therefore, it is recommended that nurses be aware of it and respond to the same early. 

STS is a form of post-traumatic stress disorder (PTSD). Symptoms include hyperarousal, avoidance, sleep disturbance, and intrusive thoughts through indirect experience of the trauma experienced by the patients, during the process of caring for the patients [8,9]. These symptoms occur acutely [8,9], have almost the same symptoms as those of PTSD, and the difference between them is classified according to the presence or absence of direct trauma [8]. Nurses experience various somatization symptoms, sleep disturbances, boredom, depression, and anxiety due to STS [10,11]. Indifference towards and fear of patients lead to an increase in false judgments and medication errors, which in turn cause an increase in turnover and decrease in nursing productivity [12].

Sleep disorders, numbness, negative emotional perception, and PTSD affected nurses who cared for severe acute respiratory syndrome (SARS) patients [13] and Middle East respiratory syndrome (MERS) patients [14], and the significant factor influencing PTSD was the experience of nursing patients with infectious diseases. Therefore, whether nursing patients with infectious diseases acted as a direct cause of trauma for the nurses or resulted in STS that occurred due to the process of caring for patients with infectious disease patients needs to be clarified; appropriate interventions and treatments are also required. However, studies on STS in nurses have mainly been conducted on emergency nurses [15,16], intensive care unit nurses [17,18], and nurses caring for patients with severe disease conditions, such as nurses in oncology wards [19,20]. On the other hand, there have been few studies on the STS experienced by nurses caring for infectious disease patients during a pandemic. 

The personal factors influencing high STS in nurses were identified as follows: being a woman [21,22], being younger in age [23,24], being unmarried [25,26], possessing low self-resilience and high empathy [24,27], and possessing a lack of active stress coping strategies. Social factors influencing the same involved a lack of social support [23,28]. Work-related factors such as caring for a patient with a severe disease state or excessive emotional work such as continuous pain or the dying of patients and caregivers may also affect STS [15,28]. Higher working hours [21], less clinical experience [29,30], and a lack of nursing staff also affect STS. STS was high when the nursing workforce and resources were insufficient [25]. In addition, emotional intelligence is the ability to accurately understand not only oneself, but also the emotions of others, and to control one’s emotions according to circumstances [31]. People with high emotional intelligence tend to positively assess stressful situations experienced and showed desirable stress coping behavior [32,33]. 

Due to the global pandemic of COVID-19, the STS of nurses caring for COVID-19 patients in the clinical field is expected to be high, and depression, anxiety, and sleep disturbance can adversely affect patient care. Previous studies have shown that the STS of nurses is influenced by factors such as stress coping strategies, social support, clinical experience, working hours, and job-related factors such as the nursing work environment. In addition, since high emotional intelligence has a positive effect on coping with stress, it is expected that there will be a difference in STS depending on the level of emotional intelligence of nurses. Since it is possible to predict the STS of a nurse, prevent it, and treat it, it is important to identify STS for nurses caring for COVID-19 patients and identify factors that affect it. Therefore, this study aimed to provide basic data for developing strategies to alleviate the STS of nurses caring for COVID-19 patients in the future by identifying the degree of STS and its influencing factors. The specific purposes were as follows:

- Determine the degree of STS of nurses caring for COVID-19 patients.
- Check the difference in STS according to the personal, social, and job-related factors of nurses caring for COVID-19 patients.
- Identify factors influencing STS in nurses caring for COVID-19 patients.

## 2. Materials and Methods

### 2.1. Study Design

**Figure 1:**
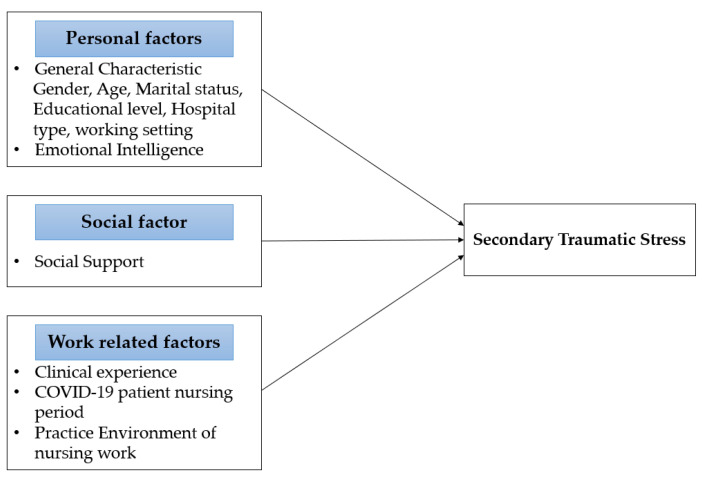
Conceptual Framework.

This study used a descriptive design to identify STS of nurses caring for COVID-19 patients, and to identify various factors that influence STS. The conceptual framework of this study is shown in Figure 1.

### 2.2. Sample

The sample size was calculated using the G*Power 3.1.9.2 program with a significance level (α) of 0.05, a medium effect size of 0.15 [34], statistical power (1 − β) of 0.80, and 11 independent variables in multiple regression; 123 participants were required. In consideration of the 10% dropout rate, a total of 136 nurses who directly cared for COVID-19 patients participated in this study. The inclusion criterion for the participants was nurses who had experience in providing direct nursing care to COVID-19 inpatients, and the exclusion criteria were nurses diagnosed with PTSD and head nurses. 

### 2.3. Measures

#### 2.3.1. Secondary Traumatic Stress (STS)

STS was measured by Stamm’s [9] Professional Quality of Life Scale (ProQOL) Version 5 of the Korean version [22]. Ten questions (numbers: 2, 5, 7, 9, 11, 13, 14, 23, 25, 28) corresponded to STS, a sub-domain of compassion fatigue. It is a questionnaire documenting fear, sleep disturbance, avoidance of confused events, and continuous thought for the last 30 days, rated on a 5-point Likert scale ranging from 1 = ‘never’ to 5 = ‘very often’, and the higher the score, the higher the STS. The reliability of STS in this study was Cronbach’s α = 0.61. 

#### 2.3.2. Emotional Intelligence

Emotional intelligence was measured by a scale modified and supplemented for nurses by adapting the Wong and Law Emotional Intelligence Scale (WLEIS), developed for workers by Wong and Law [31] in Korean [35]. The modified emotional intelligence scale consists of four aspects (self-sensibility, understanding of others, emotional control, and emotional utilization), and consists of a total of 14 questions [35]. Each question is evaluated on a five-point Likert scale ranging from 1 = ‘not at all’ to 5 = ‘very much’, and higher the score, higher the emotional intelligence. In this study, the reliability of emotional intelligence scale was Cronbach’s α = 0.63. 

#### 2.3.3. Social Support

To measure social support, we used The Multidimensional Scale of Perceived Social Support (MSPSS) designed by Zimet et al. [36], converted into Korean with proved reliability and validity [37]. The scale possessed three sub-domains on whether they are supported by family members (question numbers: 3, 4, 8, 11), friends (question numbers: 6, 7, 9, 12), and significant others (question numbers: 1, 2, 5, 10). It consists of a total of 12 questions, with 4 questions in each domain. Each question is rated on a 5-point Likert-type scale ranging from 1 = ‘not at all’ to 5 = ‘very much’, and the higher the score, the higher the social support. The reliability of social support in this study was Cronbach’s α = 0.84, and the reliability of each sub-domain was Cronbach’s α = 0.66 for support from family, Cronbach’s α = 0.68 for support from friends, and Cronbach’s α = 0.77 for support from significant others. 

#### 2.3.4. Practice Environment of Nursing Work

The Korean version of Practice Environment Scale of Nursing Work Index (K-PES-NWI) [38] designed by Lake [39] was used to measure the practice environment of nursing work, encompasses the physical environment of a hospital as perceived by an individual, interactions between colleagues, and hospital policies. The scale consists of 5 sub-domains and a total of 29 questions: 9 questions on ‘Nursing Foundations for Quality of Care’, 9 questions on ‘Nurse Participation in Hospital Affairs’, 4 questions on ‘Staffing and Resource Adequacy’, 4 questions on ‘Nurse manager Ability, Leadership, and Support of Nurses’, and 3 questions on ‘Collegial Nurse-Physician Relations’. The score is recorded on a 4-point Likert scale ranging from 1 = ‘not at all’ to 4 = ‘very much’. Higher scores indicate a better nursing work environment as perceived by the nurse. The reliability of the practice environment of nursing work in this study was Cronbach’s α = 0.89, and the reliability of ‘Nursing Foundations for Quality of Care’ was Cronbach’s α = 0.82, and that of ‘Nurse Participation in Hospital Affairs’ was Cronbach’s. α = 0.63, of ‘Staffing and Resource Adequacy’ was Cronbach’s α = 0.66, for ‘Nurse manager Ability, Leadership, and Support of Nurses’ Cronbach’s α = 0.65, and for ‘Collegial Nurse-Physician Relations’ Cronbach’s α = 0.62. 

### 2.4. Data Collection

From 5 September to 26 September 2020, an online survey was conducted on the nurses’ online community in South Korea. A questionnaire consisting of items on STS and general characteristics of the participants, personal factors, social factors, job-related factors, and explanations for participation in the study (the purpose of the study, the participants, the procedure and method of the study, expected risks and benefits, compensation for losses due to participation in the study, consent to and withdrawal of research participation, personal information protection, and publication of research results) was provided on the URL. After obtaining approval in advance by explaining the purpose of the research and the method of research to the community management team, information was collected by posting the recruitment announcement of the participants on the bulletin board. 

### 2.5. Data Analysis

Data were analyzed using IBM SPSS Statistics 27.0 for Windows. Descriptive statistics, and individual, social, and work-related factors were used to obtain frequency with percentage or mean with standard deviation. Differences in STS on individual, social, and work-related factors were identified for normality, and analyzed using the Chi-square, independent t-test, and ANOVA. Post-hoc analysis was conducted using the Scheffé test. Hierarchical regression analysis was conducted to identify various factors that influence participants’ STS, and before the regression analysis was conducted, the basic assumptions of the regression model (normality of error, linearity, equal variance, and independence) were satisfied, and the problem of multicollinearity were checked. In Model 1, general characteristics and clinical experience, patient nursing period, and hospital type were used as inputs, and in Model 2, emotional intelligence, social support, and nursing work environment were added to Model 1 to determine the magnitude of the influence of factors that affect STS and compared.

### 2.6. Ethical Considerations

The study was approved by the Institutional Review Board (IRB) of Ewha Womans University (ewha-202009-0002-01). Written informed consent was exempted by the confirmation of IRB. If the participants clicked ‘I agree’ on the first page of the online survey and filled out the questionnaires, this was considered as consent. When the recruitment for the online survey was announced, the purpose, procedure, and method of the study, privacy and confidentiality, and data storage and disposal were provided in detail to the participants, and an explanation of participation in the study was posted on the first page of the online questionnaire accessed through the URL posted. In addition, the participants were informed that they could voluntarily participate in the study and that anonymity was guaranteed. It was posted that the online survey can be terminated at any time according to the participants’ intention, and the discontinued survey data will not be used in the study data. After the survey was completed, a mobile gift voucher was provided to those who agreed to provide mobile phone numbers. Access to the collected data was restricted; only the researcher could access the data by setting a password on the researcher’s personal PC. The collected data will be discarded immediately after being stored for 3 years. 

## 3. Results

### 3.1. Differences in STS by General Characteristics

Table 1 presents differences in STS according to general characteristics. Of the participants, 92.6% were women, and the age range was from 20 s to 40 s or older. Of the participants, 121 (89%) were in their 20s and 30s, and the average age was 31.89 ± 6.40 years. Of the participants, 53% had a bachelor’s degree. There were 53 (39%) nurses working at a hospital dedicated to COVID-19, and 28 (20.6%) working at the National Safe Hospital, and the average clinical experience was 4.59 ± 3.30 years. The period of nursing for COVID-19 patients was 106.94 ± 74.55 days, with 61 participants (44.9%) having more than 30 days and less than 90 days of continuous experience. Of the total number of participants, 109 (80.1%) experienced a shortage of supplies, and 118 (86.8%) had experience in infectious disease-related education. 

There were statistically significant differences in STS by age (F = 3.078, *p* < 0.05), hospital type (F = 4.660, *p* < 0.05), clinical experience (F = 3.882, *p* < 0.05), and duration of care for COVID-19 patients (F = 5.515, *p* < 0.001). As a result of the post-hoc analysis, it was found that a nurse working in a hospital dedicated to COVID-19 infectious diseases or a hospital that does not correspond to the National Safe Hospital has higher STS than a nurse working at the National Safe Hospital. In addition, nurses with more than five years of clinical experience suffered higher levels of STS than participants with more than one year and less than three years of experience. Participants who cared for COVID-19 patients for more than 90 days and less than 180 days suffered higher STS than those who cared for COVID-19 patients for less than 90 days and more than 30 days, or less than 30 days.

### 3.2. Correlations between STS, Emotional Intelligence, Social Support, and Practice Environment of Nursing Work

A correlation was drawn between STS, emotional intelligence, social support, and practice environment of nursing work (Table 2). Emotional intelligence showed a significant positive correlation with social support (r = 0.511, *p* < 0.001), and a significant negative correlation with STS (r = −0.215, *p* =0.012). Social support showed a significant negative correlation with practice environment of nursing work (r = −0.185, *p* =0.031) and STS (r = −0.395, *p* < 0.001).

### 3.3. Factors Influencing STS

A hierarchical regression was performed to identify factors influencing STS in nurses caring for COVID-19 patients. In Model 1, age, clinical experience, duration of COVID-19 patient care, and hospital type were inputs, and in Model 2, sub-domains of emotional intelligence, social support, and practice environments of nursing work were added to Model 1, to determine the influencing factors of STS. They were identified by multiple regression analysis (Table 3). 

In order to confirm whether the basic assumptions of the regression analysis were satisfied, the normality of the residuals, linearity, equal variance, and independence of the independent and dependent variables were checked. As a result, normality and linearity were satisfied, and the Durbin–Watson value was 1.928, which was not in autocorrelation. As a result of verifying the multicollinearity between the independent variables, the tolerance in Model 1 was 0.33–0.97 and the variance inflation factor (VIF) was 1.03–3.04; in Model 2, the tolerance was 0.29–0.88, which was greater than 0.1, and the VIF was 1.13–3.54. It was determined that there was no multicollinearity problem which was smaller than 10. 

As a result of hierarchical regression analysis, the regression model of Model 1 was statistically significant (F = 6.21, *p* < 0.001), explanatory power was 16.0%, and the significant influencing factors of STS were the duration of care for patients with COVID-19 above 30 days (β = 0.28, *p* < 0.001) and an undesignated hospital related to COVID-19 (β = 0.21, *p* = 0.038). In Model 2, the explanatory power was 27.6%, and the regression model was statistically significant (F = 3.54, *p* < 0.001). In Model 2, the factor influencing STS of participants was the support of a friend in the category of social support (β = −0.21, *p* = 0.039). 

## 4. Discussion

This study was conducted to determine the degree of STS of nurses caring for COVID-19 patients, and to determine the factors that influence it. 

The participants’ STS was found to be at a moderate level with an average of 31.23 ± 5.12 points on a total score of 50 points. This was higher than an average of 27.42 ± 5.72 points in a study on intensive care unit nurses [28] and higher than the average of 28.97 ± 8.16 points in a study on emergency nurses [15]. This suggests that nurses caring for COVID-19 patients are experiencing higher STS. As COVID-19 is leading to a long-term pandemic, nurses caring for COVID-19 patients are experiencing constant and high-intensity stressful situations, so strategies to cope with STS are needed. According to the results of previous studies [40] psychological capital, such as self-efficacy and resilience, has a correlation between STS and negligence [40]; it is possible to respond positively to STS through intervention to reinforce it. Debriefing of stressful situations or traumatic events experienced during the period may be used [41]. In addition, after implementing the Mindful Self-Compassion (MSC) program for 8 weeks on nurses, STS was significantly reduced compared to pre-implementation, and negative mental and psychological symptoms such as distress, burnout, anxiety, and depression were reduced [42]. In the present pandemic, it is critical to establish a support system, such as a mindfulness program, for the STS-management of nurses caring for COVID-19 patients.

In this study, STS was significantly higher in participants with more than five years of clinical experience compared to those with one to three years of clinical experience, contrary to a prior study which indicated that STS was higher among those with less clinical experience [29,30]. Considering that this is a pandemic, it is possible that this is the result of an increased work burden on nurses with more experience, due to a lack of workforce or resources, and because they are more likely to be exposed to stressful situations. However, it is necessary to consider the same from multiple perspectives. 

In this study, it was found that a period of nursing COVID-19 patients for 30 days or more had a significant effect on nurses’ STS. A study of healthcare workers caring for COVID-19 patients [43] found that nurses were at higher risk of stress, depression, anxiety, and burnout, than other healthcare workers. As the anxiety level was higher at 10 months than at 5 months, the period of caring for COVID-19 patients may be associated with a higher level of stress. 

In addition, when nursing COVID-19 patients, the time the nurses are needed approximately doubled as the work intensity increased due to using various personal protective equipment through negative pressure isolation [3]. This continuous increase in work intensity can negatively affect nurses’ STS. Therefore, it is necessary to recognize and manage the situation of nurses exposed to extreme stress early in the situation where COVID-19 continues for a long time, and a multi-faceted solution to solve the intensity of nurses’ work is required. 

In this study, if the nurses were caring for COVID-19 patients for more than 30 days, it had a significant effect on nurses’ STS. Therefore, it is necessary to manage human resources appropriately, such as limiting the working period of nursing COVID-19 patients to less than 30 days. However, since there may be differences in the intensity of work and stress depending on the level of a COVID-19 outbreak, the work should be adjusted, not only by considering the working period but the work intensity according to the intensity of the COVID-19 outbreak. 

There was a difference in the STS depending on the type of hospital in which the participants worked, and it was found that nurses working in hospitals not dedicated to COVID-19 infectious diseases or not designated as National Safe Hospitals had nurses experiencing higher STS than nurses working at National Safe Hospitals. The hospital dedicated to COVID-19 infectious diseases is a nationally designated hospital that treats COVID-19 infected patients who need hospitalization and the Korean government is supporting workforce, facility costs, and equipment costs [44]. In a National Safe Hospital, respiratory disease patients receive treatment at a selective clinic. In addition, when suspected COVID-19 patients enter the hospital, they are placed in a single occupancy room or in a one-bed room [45]. According to previous studies [46,47], the degree of STS of nurses caring for COVID-19 patients was lower than that of nurses in general wards. This is because nurses caring for COVID-19 patients are more focused on the positive outcomes of patient care than on their own emotional stability, and because they know more about COVID-19, meaning they emotionally endure the situation better. In the case of nurses working in hospitals not designated nationally for COVID-19, the ability to cope with stressful situations or adaptability decreases because they lack sufficient equipment and training for COVID-19, and patients with COVID-19 are nursed in unexpected situations. This can lead to higher STS of participants. 

There were no significant differences between the participants’ experience of lack of supply of medical goods/equipment and STS, but approximately 80% of the participants experienced insufficient supply of medical goods/equipment. According to a previous study, the lack of PPE kits, beds, ventilators, and the number of nurses could increase the burden of STS for nurses caring for COVID-19 patients [48]. These results suggest that sufficient human support and resources are needed in a nursing environment for COVID-19 patients, and insufficient support may have an effect on increasing the degree of STS, especially if it is not a COVID-19 National Safe Hospital.

Social support of participants was identified as a factor that significantly influenced STS. It has been confirmed in previous studies that lower the social support, higher the STS of nurses, especially when there was no supportive colleague in the workplace [22,23,27]. This suggests that the social support system is important. During the COVID-19 pandemic, a nurse who works will avoid seeking help from others due to concerns about the transmissibility of the COVID-19 infection not only for themselves, but also for family and friends, which is associated with low social support [49]. Higher social support can have a positive impact on the emotional wellbeing of nurses caring for COVID-19 patients who are experiencing various stressful situations [48]. It is necessary to establish a social support system for the nurses caring for COVID-19 patients. However, in the present COVID-19 situation, nurses wear protective equipment and perform patient care in isolation rooms alone, so they experience limited communication with colleagues, work support, and teamwork activities. Therefore, hospitals should help alleviate the emotional difficulties of nurses caring for COVID-19 patients by preparing measures to strengthen social support, such as sharing work experiences among colleagues, supporting empathy, and mentoring. Social support reportedly has a direct effect and a buffer effect that protects individuals from the harmful effects of stress [50,51]. It is also an important variable in reducing symptoms of STS and burnout in nurses [52]. In addition, according to a previous study, it is possible that STS plays a mediating role in the association between social support and burnout among nurses [53]. Through further research, it is necessary to find out whether social support has a buffer effect between STS and symptoms of nurses caring for COVID-19 patients. In addition, the support of human capital and resources for nursing COVID-19 patients mentioned above is one of the aspects of social support that requires special attention. 

In this study, participants’ emotional intelligence showed a significant negative correlation with STS, but the causal relationship was not significant. A study [5] on nurses caring for COVID-19 patients found that a higher emotional intelligence had a negative correlation with negative emotions such as distress or depression. Also, it was thought that emotional intelligence could act as a buffer against the negative effects of stress [54] In this study, the causal relationship between the participants’ emotional intelligence and STS was not significant, and there are insufficient studies to support this. It is necessary to understand whether emotional intelligence can be used as a buffer for STS. 

Although this study is meaningful in that it reports the factors influencing the STS of nurses in a pandemic situation, there are some limitations. In this study, in order to identify the factors influencing the STS of nurses caring for COVID-19 patients, work-related characteristics, COVID-19 patient nursing period, and experiences with shortage of supplies, were investigated through a self-report questionnaire. Since an online survey was conducted due to the risk of infection of COVID-19, errors may have occurred in data collection. In addition, depending on the department, the degree of STS may be different due to the difference in work intensity due to the severity of the patient’s condition and the number of patients per work. Considering that the COVID-19 situation continues for more than a year, it is important to identify personal, social, and work-related factors that affect STS according to the trend of COVID-19 outbreaks for nurses caring for COVID-19 patients. Therefore, further research about the topic is necessary. 

## 5. Conclusions

The nurses caring for COVID-19 patients experienced moderate STS; it was found that STS differed depending on the length of clinical experience, the type of hospital, and the availability of social support. Considering that the nurses caring for COVID-19 patients during a pandemic experience persistent and high-level stress situations, it is necessary to identify the factors that influence STS of nurses caring for COVID-19 patients. Programs using psychological capital or measures such as debriefing can help to employ positive coping strategies to combat STS. Further, it is necessary to adjust the work in consideration of the intensity of work according to the level of the COVID-19 outbreak. In addition, there is a need to establish a social support system to alleviate the emotional difficulties of nurses caring for COVID-19 patients, including providing sufficient human and material resources, and the education necessary to care for COVID-19 patients. 

## Figures and Tables

**Table 1:** Differences in secondary traumatic stress by general characteristics (N = 136).

Variables	Categories	*n* (%)	M ± SD	Secondary Traumatic Stress	
				M ± SD	t/ F (*p*) Post-hoc
Gender	Male	10 (7.4)		32.40 ± 6.77	0.75 (0.454)
	Female	126 (92.6)		31.13 ± 4.99	
Age (years)	20–29	60 (44.1)	31.89 ± 6.40	30.05 ± 6.05	3.08 (0.049)
	30–39	61 (44.9)		32.00 ± 4.10	
	40≤	15 (11.0)		32.80 ± 3.91	
Marital status	Not married	78 (57.4)		30.56 ± 5.87	1.55 (0.295)
	Married	55 (40.4)		32.07 ± 3.62	
	Others	3 (2.2)		33.00 ± 7.00	
Educational level	College	15 (11.0)		31.20 ± 4.57	1.50 (0.228)
	University	72 (53.0)		30.57 ± 6.12	
	Graduate school	49 (36.0)		32.20 ± 3.26	
Hospital type	COVID-19 Infection Dedicated Hospital ^1^	53 (39.0)		30.41 ± 4.82	4.66 (0.011) 2 < 3
	National Safe Hospital ^2^	28 (20.6)		29.71 ± 6.44	
	Undesignated Hospital for COVID-19 ^3^	55 (40.4)		32.78 ± 4.27	
Work setting	Intensive Care Unit	24 (17.6)		33.00 ± 5.34	0.96 (0.434)
	Medical Unit	69 (50.7)		30.96 ± 4.97	
	Surgical Unit	28 (20.6)		30.93 ± 5.24	
	COVID-19 Dedicated Unit	4 (2.9)		30.75 ± 8.38	
	Others	11 (8.1)		30.00 ± 3.90	
Clinical experience (year, *n* = 135)	<1 ^1^	5 (3.7)	4.59 ± 3.30	33.40 ± 9.37	3.88 (0.011) 2 < 4
	1≤ <3 ^2^	36 (26.7)		29.19 ± 5.44	
	3≤ <5 ^3^	32 (23.7)		30.75 ± 4.66	
	≥5 ^4^	62 (45.9)		32.55 ± 4.39	
COVID-19 patient nursing period (day)	<30 ^1^	6 (4.4)	106.94 ± 74.55	24.17 ± 6.43	5.52 (0.001) 1 < 2,3
	30≤ <90 ^2^	61 (44.9)		31.66 ± 4.08	
	90≤ <180 ^3^	39 (28.7)		32.41 ± 5.60	
	≥180 ^4^	30 (22.1)		30.23 ± 5.08	
Experiences with shortage of supplies	Yes	109 (80.1)		31.61 ± 5.16	−1.78 (0.077)
	No	27 (19.9)		29.67 ± 4.74	
Experiences with infectious disease-related education	Yes	118 (86.8)		31.48 ± 5.06	−1.49 (0.138)
	No	18 (13.2)		29.56 ± 5.38	

**Table 2:** Correlations among secondary traumatic stress, emotional intelligence, social support, and practice environment of nursing work (N = 136).

Variables	Emotional Intelligence	Social Support	Practice Environment of Nursing Work	Secondary Traumatic Stress
	r (*p*)			
Social support	0.51 (<0.001)			
Practice environment of nursing work	−0.09 (0.302)	−0.19 (0.031)		
Secondary traumatic stress	−0.22 (0.012)	−0.40 (<0.001)	−0.01 (0.941)	
Mean ± SD	47.88 ± 5.80	42.99 ± 7.80	72.21 ± 13.23	31.23 ± 5.12
Min-Max	26–63	16–60	37–109	17–48

**Table 3:** Influencing factors on secondary traumatic stress (N = 136).

Variables	Model 1				Model 2			
	B	β	*t*	*p*	B	β	*t*	*p*
Constant	230.08		70.42	<0.001	380.57		60.69	<0.001
Age	−0.02	−0.02	−0.14	0.889	−0.12	−0.15	−10.02	0.309
Clinical experience	0.02	0.17	10.37	0.174	<0.02	<0.18	10.47	0.143
COVID-19 patient nursing period ≥30 days (<30 days = ref.)	60.81	0.28	30.37	0.001	50.97	<0.24	20.92	0.004
Hospital type: Undesignated hospital for COVID-19 (COVID-19 Safe hospital = ref.)	20.15	0.21	20.10	0.038	20.27	0.22	20.15	0.033
Emotional intelligence					−0.01	−0.01	−0.09	0.926
Social support								
Support of family					0.02	0.01	0.11	0.916
Support of friends					−0.34	−0.21	−20.08	0.039
Support of others					−0.22	−0.14	−10.20	0.232
Practice environment of nursing work								
Nursing foundations for quality of care					0.10	0.11	0.77	0.444
Nurse participation in hospital affairs					−0.09	−0.06	−0.54	0.591
Staffing and resource adequacy					−0.29	−0.15	−10.35	0.180
Nurse manager ability, leadership, and support of nurses					−0.29	−0.15	−10.35	0.181
Collegial nurse-physician relations					0.29	0.12	10.16	0.250
	R^2^ = < 0.16, _adj_R^2^ = < 0.13 F = 60.21, *p* < 0.001				R^2^ = < 0.28, _adj_R^2^ = < 0.20 F = 30.54, *p* < 0.001			

## Data Availability

The data presented in this study are available on request from the corresponding author. The data are not publicly available due to the information contained that could compromise the privacy of research participants.
